# Sterilisation of Catheters1A lecture delivered before the School of Nurses, Buffalo Hospital of the Sisters of Charity, November 8, 1899.

**Published:** 1900-02

**Authors:** Byron H. Daggett

**Affiliations:** Buffalo, N. Y.


### Lecture.

### STERILISATION OF CATHETERS.[1]

By BYRON H. DAGGETT, M. D., Buffalo, N. Y.

THE late Dr. William Pepper addressed a session of the International Medical Congress which met in Washington twelve years ago, said: "That medicine had advanced more in the past twenty years than in the previous two thousand."

Its most notable advancement is in the development of bacteriology. The discovery and demonstration, that bacteria or their toxins are the causes of some of the most active and virulent diseases, followed the claim and demonstration made by Pasteur, in 1860 that there was no development of germs in organic infusions sterilised by boiling, and that fermentation and putrefaction depended upon the introduction of living germs into such infusions, and thus he settled forever the question of spontaneous generation.

Microbes are coëxtensive and coëval with men. Microbes virulent and beneficent exist in multitudinous hosts; are constantly assaulting the citadel of our homes, incessantly active, bearing either weal or woe. They became virulent in debased civilisation, over crowding, filth and unsanitary conditions of the cities of the ancient continent. Microbic diseases are spread by human intercourse and their ravages must be met and subdued by isolation, quarantine, disinfection and sterilisation.

> "We have boiled the hydrant water,
> We have sterilised the milk,
> We have strained the prowling microbe
> Through the finest kind of silk;
> We have bought and we have borrowed
> Every patent health device,
> Now, Dr. Wilson tells us,
> We have got to boil the ice."

Devaine demonstrated the etiological (causal) relation of germs to specific diseases. Following this demonstration a host of busy workers in bacteriology made numerous important discoveries. The anthrax bacillus was found in the blood of animals suffering from this malady and by experimental inoculations, this germ was shown

1. A lecture delivered before the School of Nurses, Buffalo Hospital of the Sisters of Charity, November 8, 1899.

to be the cause of anthrax. A germ (spirellum) was found in the blood of a patient suffering from relapsing fever. The typhoid bacillus was found by Eberth and Koch in 1888 and in the same year Sternberg demonstrated the presence of a specific germ in croupus pneumonia. In 1882 Koch announced the discovery of the tubercle bacillus, the recognised cause of consumption. Our knowledge of pathogenic bacteria is rapidly growing.

These discoveries demonstrate: (1) That contagious diseases are caused by microbes; (2) that contagious diseases produce microbes, which either as microbes or their products beget diseases peculiar to themselves, in those who are well.

The practice of surgery and obstetrics has been revolutionised by the revelations of bacteriology. If infection be localised and confined to accessible areas, it can be successfully combatted by germicides, but given internally in sufficient potency and quantity to destroy bacteria, they will destroy life itself.

With this introduction to the general subject I pass to the lesson of the day, namely,

### STERILISATION OF CATHETERS.

There are several reasons for giving special attention to urinary asepsis and sterilisation of urinary instruments: (1) The urinary tract is lined by a very fragile and delicate membrane, which is easily eroded or broken, opening a point for infection; (2) The urethral canal is the constant habitat of bacteria; (3) the secretion from the kidneys, often bearing bacterial infection ready for the fray, drenches the passage way several times each day; (4) infection may be conveyed into the urethral canal by catheters and other instruments; (5) sterile instruments in the hands of the unskilful may open the way for infection by agents already at hand.

Previous to the introduction of aseptic methods all wounds developed more or less infection and resulting suppuration. During my student days the professors taught that laudable pus was a harbinger of the healing process. We now know that pus is a harbinger of infection and that infection is neither laudable nor commendable. One professor said that pus was dead blastema; another, that it was not blastema dead or alive.

Aseptic surgery was unknown during the Civil War, and all wounds suppurated and many developed gangrene. As a United States Pension Surgeon I saw scores of large, deeply depressed and attached scars caused by gangrenous wounds. How many died from wound infection no man will ever know. In the recent Spanish War these conditions were obviated by aseptic methods, notwithstanding the heat of a torrid zone, exposure and the presence of the accumulated filth of centuries.

Previous to the introduction of asepticism, catheter cystitis was a frequent complication of gynecological operations; now, under strict aseptic and antiseptic rules it is rare.

As to instruments, the soft or nonmetalic are preferable, for the reason that there is less power or liability to produce injury of the lining membrane. The objection to the soft instrument is, that sterilisation is more difficult than with the metalic catheter. As a rule, the soft instrument should be chosen. The rubber and woven catheters call for a special method of sterilisation by reason of their composition. The best of them will not endure boiling but for a few minutes; and, as a rule, the chemical germicidal agents quickly destroy them. They decay rapidly in moisture, heat, various fumes, oils and all fatty substances. The best catheters are smooth, both on the inner and outer surfaces, and have no crevices or depressions for the lodgement of infection.

The first step is to wash the catheter with soap suds, outer surface with scrubbing, inner surface by forcing the suds through its lumen with a syringe. Then dry it at a temperature of 100° Fahr.

Guyon, of the Neckar Hospital, Paris, uses dry heat at a temperature of 140°C. The instruments must be perfectly dry, must not come in contact with each other, or the bottom of the oven. This process would cook, the usual run of soft catheters. A good quality of gum and rubber catheters may be placed in boiling water, for not more than five minutes; still the best of them, will not last long under this treatment. Sufficient exposure to steam, to sterilise both gum, rubber and woven catheters will destroy them. Alcohol, solutions of carbolic acid, soda and corrosive sublimate will destroy soft catheters.

Wolf divides septic catheterisation into three stages : (1) Sterilisation of the instruments; (2) maintaining sterility when not in use; (3) aseptic introduction. He also alleges that it is difficult to sterilise nonmetalic catheters without injuring the fabric. Formalin, 1 per cent. solution, is effectual and harmless, but painful to the patient. He claims that 1 per cent. aqueous solution of corrosive sublimate, in equal proportion of glycerine, fulfills all the requirements and that this compound will sterilise a catheter after six hours immersion or five minutes boiling.

Mansel Moullin, of London, says that aseptic catheterism is one of the problems of modern surgery. Dr. Moullen's plan is to disinfect the catheter by boiling, and prepare the hands and parts as if for a surgical operation and thoroughly irrigate the urethra. If the catheterisation is done several times each day, it is not necessary to use this technique but once each day. The catheters are boiled every day and placed in glass tubes, closely stopped. As soon as used each catheter is placed in a solution of boracic acid, there remains until the daily boiling takes place. After boiling and drying they are put into a dry case and tightly closed until ready for use again.

Dr. Edward Martin of the University of Pennsylvania, alleges that the medicated soaps are no better than the ordinary green soap. Exposure to mercurial vapor for twenty-four hours was effectual.

Sterilisation by paraform proposed by Janet is effectual, but paraform is very irritating to the urethral mucous membrane, and the instrument must be thoroughly washed before using.

Efforts have been made to incorporate germicides with lubricants of sufficient potency to destroy urethral bacteria, but they have been failures. A good plan for cleansing the urethra is to attach to the tube of a fountain syringe and flush the urethra as the catheter is introduced. This method not only cleanses the urethra, but also distends and renders it less liable to trauma or injury. In Bellevue Hospital, the catheter is thrown immediately after using into boiling water, then washed with hot soap suds and boiled. Dr. Alexander, New York, has ceased the use of urethral sterilisers before catheterisation, but is careful to cleanse the meatus. It seems curious that we get such diverse results from catheterisation—some persons are readily infected, while others appear to be more or less immune to infection.

In the presence of pus and congestion or inflammatory conditions of the urethral tract, there is much greater liability of infection. For this reason special care should be taken to avoid force or traumatism and to apply flushing with antiseptic, detergent fluids. Over distension of the bladder, begets vascular engorgement, so that, catheterisation should be systematically and regularly carried out to lessen this source of infection.

Brower, St. Louis, says, so long as urine is passed with pathologic force, just so long will we be unable to cure chronic inflammatory conditions. If force aggravates pathological processes so it will beget them. It is never necessary, for if the catheter is blocked, it is due to improper application oı an improper instrument. Never be hasty. Nature will aid you, unless you thwart her efforts. The catheter should be of fair size, sufficient to easily fill the urethral lumen, and its presence in the deeper urethra will cause a retroperistalsis, a vermicular action which will draw the instrument bladderward. This phenomena was noted by McNamara, of Dublin, thirty years ago, also by Civiale, who called it "swallowing backward." The pressure of any substance, in the deep or posterior urethra, will incite this movement, and utilise this principle. The bladder may be flushed without a catheter and without force, providing the body be placed flexed, in a semi-dorsal position.

Morris, of London, and other authorities, say that irrigation is harmful in the presence of acute cystitis, which I will grant, if the forced or catheter methods are used. There is another method, forceless and catheterless, which is the treatment par excellence for cystitis and infection. Agents of infection are always present in the urethral tract and we must ever be on guard against their assaults.

The use of the soft catheter has been even a more important advance in avoiding cystitis than sterilisation itself. Metalic catheters, by the traumatism they often produce, open the gates for infection, in spite of all known methods of combating them.

Various appliances have been proposed from time to time for sterilising urethral instruments; by steaming, by fumigating, boiling, and dry heat—each one advocated by its designer. Their use, outside of a hospital, is not practical and it has not been shown that they are more efficient than the ordinary methods by boiling, steaming, sudsing, washing in solutions of the usual germicidal drugs and drying.

The condition of the urine is a matter for consideration, when using catheters. The chemical reaction of the urine is a guide to urinary therapeutics, and the remedies for combating urinary sepsis. The urine should be held slightly acid, its normal condition by phos. soda. The chemical reaction of this fluid, is one of the questions the doctor will often ask before prescribing, and before passing a catheter.

Various remedies are given for the purpose of making the reaction normal and to render or maintain the urine as aseptic as possible : Salol, eucalyptus, oil of wintergreen, or salicylic acid or its salts, benzoate of soda, benzoic acid, and the sodas or potassium or ammoniacal compounds, kava kava, sandal oil, triticum, parira brava, and many others.

The condition of the urethra and of the urine, especially in cases requiring repeated catheterisation, and in wards where many patients require this service, should be known. The care of catheters should also be double guarded under these circumstances.

Johnson & Johnson, manufacturers of soft catheters, direct that their instruments should never be coiled, and should be kept in packed cases to protect them from dust and dirt. They should not be powdered or greased. After use they should be flushed with hot water, but not soaked. Their lumen should be thoroughly cleaned and then thoroughly dried. Flush with 1 per cent. of formalin to sterilise before and after using, both inside and outside. Wash off this solution with pure water, to prevent irritation of the membrane. Soft catheters should not be immersed for any length of time in any fluid. No soft catheter, as a rule, should be used more than once each day.

There are several forms of microbes inhabiting the urinary passages, and others may be introduced by instrumentation and by the circulation—some are virulent, others are benign. Their names and classification need not concern us—for sterilisation they are all alike. As a rule, use the soft catheter, woven or rubber. The long soft instrument is very useful in catheterisation of the female patients, to avoid dripping, and is especially useful in washing the bladder. If the urine is turbid or there is any indication for washing the bladder, this should be done before the withdrawal of the instrument. For this purpose use sterilised water, chloral, boracic acid, glycerine and ichthyol, or a weak solution of some form of silver. Use mild, non-irritating remedies lest they induce rhythmic action of the bladder in the presence of urinary retention, frequent and painful urination or when employing the catheterless method. It is often good practice to flush out the bladder following catheterisation. Chloral hydrate, 10 to 15 grains to the quart of water 1-10 to 1-12 per cent. I often use a combination of chloral and silver nitrate for cystitis; these remedies are chemically incompatible, but practically useful.

Select your instrument and clean it, then flush the urethra, at least with a pledget of absorbent cotton wipe and clean the urethral orifice. As a routine practice, this is sufficient. My practice is to inject into the urethra, before catheterisation or sounding, a 2 per cent. solution of antipyrin. This drug is analgesic and antiseptic. It first produces a sharp tingling—say to the patient it will last but a moment. Then lubricate the catheter, and pass it into the bladder. If irrigation of the bladder is indicated, place the nozzle of a small funnel into the catheter and pour the antiseptic solution into the funnel. You will not get much into the bladder, but it will be sufficient. This process is like that of washing out the stomach. In this way you use no force.

The sensitive, inflammed bladder resents the impact of a stream or jet from a syringe. The bladder will resent the presence of any substance, however bland, except in small quantity, until tolerance is established by repeated use. Remove the catheter, and before you lay it down, start it on its road to sterilisation by dipping it into hot water, either medicated or plain. In regard to lubricants, fatty substances are usually recommended, such as vaseline, oil, and various compounds of glycerine and soap. Grease or animal fat and mineral oils are not normal lubricants of mucous surfaces. Mucous secretions do not readily unite or diffuse with grease or oleaginous mixtures; mucilaginous substances, however, possess a natural affinity for mucous secretion and their union forms a smooth and almost frictionless menstruum. This quality of a mucilaginous compound may be demonstrated in the use of a sound or bougie for dilating a stricture, since an instrument one or two sizes larger may be employed with mucilage as a lubricant than when grease is used. Another point in favor of the mucilaginous lubricant is that the instrumeut is more readily cleaned when mucilage is used. Rinsing in hot water will remove this substance, while to cleanse it of the grease, requires hot soap suds and friction or rubbing. Again grease vitiates the integrity of all elastic and lacquer covered instruments, while mucilage is harmless, if not beneficial. Several formulæ have been proposed for compounding mucilaginous lubricants.

Gouley (*New York Medical Journal*, November 4, 1899,) states that web and rubber catheters are much injured by fats of all kinds, by glycerin and vaselin, and that the best lubricant is soap, deprived of glycerin and free alkali, and mixed with mucilage of chondrus crispus. He recommends the following formula :

| | |
|---|---|
| ℞—Powdered white castile soap . . . . . . . . . | 1 ounce. |
| Water . . . . . . . . . . . . . . . . | 3 ounces. |
| Mucilage of chondrus crispus . . . . . . . . | 3 ounces. |
| Formalin (40 per cent.) . . . . . . . . . . . | 10 minims. |
| Thymol . . . . . . . . . . . . . . . . . | 5 grains. |
| Oil of thyme . . . . . . . . . . . . | 5 minims. |
| Alcohol . . . . . . . . . . . . . . . . . | 15 minims. |

Mode of preparation : Heat the soap and water, and stir until a homogeneous slime is formed; then add the mucilage (one ounce of chondrus crispus to one pint) of water ; when cool pour in the formalin, then the thymol and oil of thyme mixed, with the alcohol, stir, strain and keep in a covered vessel until all air-bubbles have vanished. The result is an opalescent substance of the consistence of honey, which should be put up at once in two-ounce collapsible tubes and sterilised.

Ruggles proposed the following formula:

| | |
|---|---|
| ℞—Gum tragacanth . . . . . . . . . . . . . | gr. xlviii (48). |
| Ac. carbolic (95 per cent.) . . . . . . . . | m. ii. |
| Glycerin . . . . . . . . . . . . . . . . . | ʒiii. |
| Aqua ad . . . . . . . . . . . . . . . . | ℥iv. |

The basis of the "lubi-chondrin" is Iceland moss. Its formula is not given.

The pharmacist of the Buffalo Hospital of the Sisters of Charity constructs a lubricant from chondrus crispus as a base which is very cheap and most excellent. One who has used the properly prepared mucilaginous lubricant will never wish to use grease. Never put instruments or fingers into the jar of lubricant, always pour out sufficient for temporary use into a small plate or ointment jar lest you dirty or infect the source of supply. "A little leaven will leaveneth the whole lump."

Regarding the sterilisation of urethral instruments, metalic instruments are readily sterilised by boiling, steaming and in other ways, which are destructive to elastic catheters and bougies, so that other methods must be used

I will close by giving you my routine method for cleansing urethral instruments. Assuming they are clean when used, after use, and before I lay them down, I dip into boiling or steaming water, then into a jar of odorless naphtha or gasoline, so that they are already well cleaned. After the catheter is placed or dipped into the gasoline, put the finger over its distal end and as you withdraw it, its lumen is partially filled with the gasoline, invert the instrument and this liquid runs out, thus you have reached both out and inside of the catheter. Before putting the instrument away you may repeat this procedure, or apply some other one. When satisfied that it is clean roll it up in naphthalated gauze and over all a wrap of oiled silk.

Odorless gasoline is cleansing and drying as it rapidly evaporates. Instruments will not rust after its use if they are clean, especially if first dipped into hot water. The use of gasoline in this way will prolong the life of gum catheters and bougies. But these instruments must not be left in this fluid any length of time, since it will soften and macerate them. Soft instruments should be rubbed and polished with fine cloth or chamois skin occasionally. Gasoline is very destructive to insect life. To what extent it acts as a germicide I am unable to state, except from use as detailed above. It is very cheap, convenient and fulfils other indications and as a cleanser if not an ideal steriliser of instruments. I have used it daily for three or four years.

Armed with a bottle of chondrus crispus co., a bottle of odorless gasoline, a solution of antipyrin, some pledgets of antiseptic cotton and an assortment of catheters, wrapped in naphthalated gauze, you are thoroughly well equipped.

Eternal vigilance is the price of asepticism; even at that price you cannot always procure the results for the reason that our methods are not yet perfect.

